# Phagocytosis of Bacteria Adhering to a Biomaterial Surface in a Surface Thermodynamic Perspective

**DOI:** 10.1371/journal.pone.0070046

**Published:** 2013-07-19

**Authors:** Joana F. da Silva Domingues, Henny C. van der Mei, Henk J. Busscher, Theo G. van Kooten

**Affiliations:** Department of Biomedical Engineering, W.J. Kolff Institute, University Medical Center Groningen and University of Groningen, Groningen, The Netherlands; Universitätsklinikum Hamburg-Eppendorf, Germany

## Abstract

Bacterial biofilms can increase the pathogenicity of infection and constitute a major problem in modern health-care, especially on biomaterial implants and devices. Biofilms are difficult to eradicate by the host immune system, even with antibiotics, and have been the number one cause of biomaterial implant and device failure for decades. Therefore, it is important to understand how immune cells interact with adhering pathogens. This study firstly aims to develop a simple method to quantify phagocytosis of six different strains of staphylococci adhering on a surface with phase-contrast-microscopy. Phagocytosis of adhering staphylococci to a glass surface by phagocytes was quantified in a parallel plate flow chamber, and expressed as a phagocytosis rate, accounting for the number of adhering staphylococci initially present and for the duration of phagocytosis. Murine macrophages were more effective in clearing staphylococci from a surface than human phagocytes, which require differentiation from their monocyte or promyelocytic state during an experiment. Direct visualization of internalization of a GFP-modified *S. aureus* strain inside phagocytes confirmed the validity of the method proposed. As a second aim, the differences in phagocytosis rates observed were investigated on a surface thermodynamic basis using measured contact angles of liquids on macroscopic lawns of staphylococci and phagocytes, confirming that phagocytosis of adhering pathogens can be regarded as a surface phenomenon. In addition, surface thermodynamics revealed that phagocytosis of adhering pathogens is determined by an interplay of physical attraction between pathogens and phagocytes and the influence of chemo-attractants. For future studies, these results will help to place *in vitro* experiments and murine infection models in better perspective with respect to human ones.

## Introduction

Bacterial pathogenicity often increases when bacteria transform from a planktonic to an adhering state and start growing into a biofilm [1]–[3]. In a biofilm community, bacteria live surrounded by a heterogeneous, exopolymeric matrix, which yields protection against environmental stresses, host immune defenses or antibiotics [1], [3]–[6]. Bacterial strains vary in their ability to protect themselves against external threats by releasing extracellular polymeric substances, different exotoxins and elastases or expressing different outer membrane proteins [5], [7]–[9].

Bacterial biofilms occur both in health and disease. In modern medicine, biomaterial-associated-infection is the number one cause of implant failure [2]–[4]. In many cases of biomaterial implant failure, such as of orthopedic joint prostheses, pacemakers or vascular grafts, bacteria of the *Staphylococcus* genus are responsible for increased morbidity and mortality at high costs to the health care system [1], [3], [5]. *Staphylococcus aureus* strains are generally considered to be more virulent to the host than *Staphylococcus epidermidis*, since *S. aureus* strains produce more toxins and tissue-damaging exo-enzymes than *S. epidermidis* [5], [9]–[11].

In the host, different immune cells are recruited to an infection site to become involved in the elimination of pathogens [12]–[14]. The first cells arriving at the infection site are neutrophils and macrophages [13]. Although neutrophils are crucial in the first hours of host response to an infection, they disappear within a day [13], [14]. Macrophages then become the prevailing cells and remain at the infection site in a high concentration for several weeks. During this period, macrophages play an important role in wound healing and orchestrating the inflammatory response. After recognition and phagocytosis of pathogens, macrophages activate cellular functions such as cell proliferation and secretion of enzymes, reactive oxygen and nitrogen species, cytokines, chemokines, and growth factors, among other biological substances, to destroy the phagocytized bacteria [2], [15]. However, in the presence of a biomaterial, the normal host response is often impaired which contributes to the virulence of biomaterial-associated-infections [3], [16]–[18].

Phagocytosis of bacteria adhering on biomaterials surfaces depends amongst others, on the bacterial species involved and the affinity of the phagocyte for the bacterial cell surface and the surrounding medium [19]–[21]. Accordingly, phagocytosis can be regarded as a surface phenomenon [21] that can be analyzed on a similar surface thermodynamic basis as bacterial adhesion to a biomaterial surface. Initial interactions between two biological surfaces, as of a phagocytic cell and a bacterium, depend upon long-range, attractive Lifshitz-Van der Waals forces and upon closer approach upon short-range acid-base interactions, which can either be attractive or repulsive [4], [22]–[24]. Surface thermodynamics enables to estimate the relative contributions of Lifshitz-Van der Waals and acid-base forces in phagocytosis, based on contact angles with liquids on macroscopic lawns of phagocytic cells or bacteria prepared on membrane filters. Using measured contact angles with liquids, interfacial free energies of adhesion between phagocytes and bacteria can be calculated to predict whether phagocytosis, or strictly speaking adhesion, will be thermodynamically favorable (negative free energy of adhesion) or unfavorable (positive free energy of adhesion) [4], [6]. Light microscopy has been used to evaluate the interaction between phagocytes and other cells or bacteria but mostly in a qualitative way by observing the removal of a biofilm from a surface over time [9], [25] or using radiolabeled bacteria [26]. However, understanding the mechanisms governing phagocytosis of bacteria adhering on a biomaterial surface requires a simpler and more quantitative *in vitro* method.

In this paper, we present a simple method to quantify phagocytosis of bacteria adhering on a surface with phase contrast microscopy. The method was validated for a fluorescent staphylococcal strain using Confocal Laser Scanning Microscopy (CLSM). Subsequently, phagocytosis of six different staphylococcal strains by one murine line of macrophages, one human line of monocytes and a promyelocytic cell line were analyzed on a surface thermodynamic basis to demonstrate a relation between the rate of bacterial phagocytosis and the interfacial free energy of adhesion between the different phagocytes and bacteria.

## Results

### Quantification of Bacterial Phagocytosis by Different Phagocytes

Phagocytosis of staphylococci by different phagocytic cells on a glass substratum was quantified by enumerating the number of adhering staphylococci on the substratum over 2 h, in the absence and presence of phagocytes in a parallel plate flow chamber. The number of staphylococci phagocytized was subsequently taken as the reduction in the number of adhering bacteria (“indirect method”, see supplementary information, Figure S1 and Video S1). Since this method is based on phase contrast microscopy, it can be applied to different strains and species, including clinical isolates on transparent (bio)materials. In order to validate the results and also directly demonstrate phagocytosis, green fluorescent staphylococci were allowed to adhere and exposed to different phagocytes after which the number of staphylococci inside single phagocytic cells was enumerated using CLSM (“direct method”) and compared with results from indirect quantification. Note, that the direct method is only applicable for molecularly-engineered fluorescent strains.

**Figure 1:**
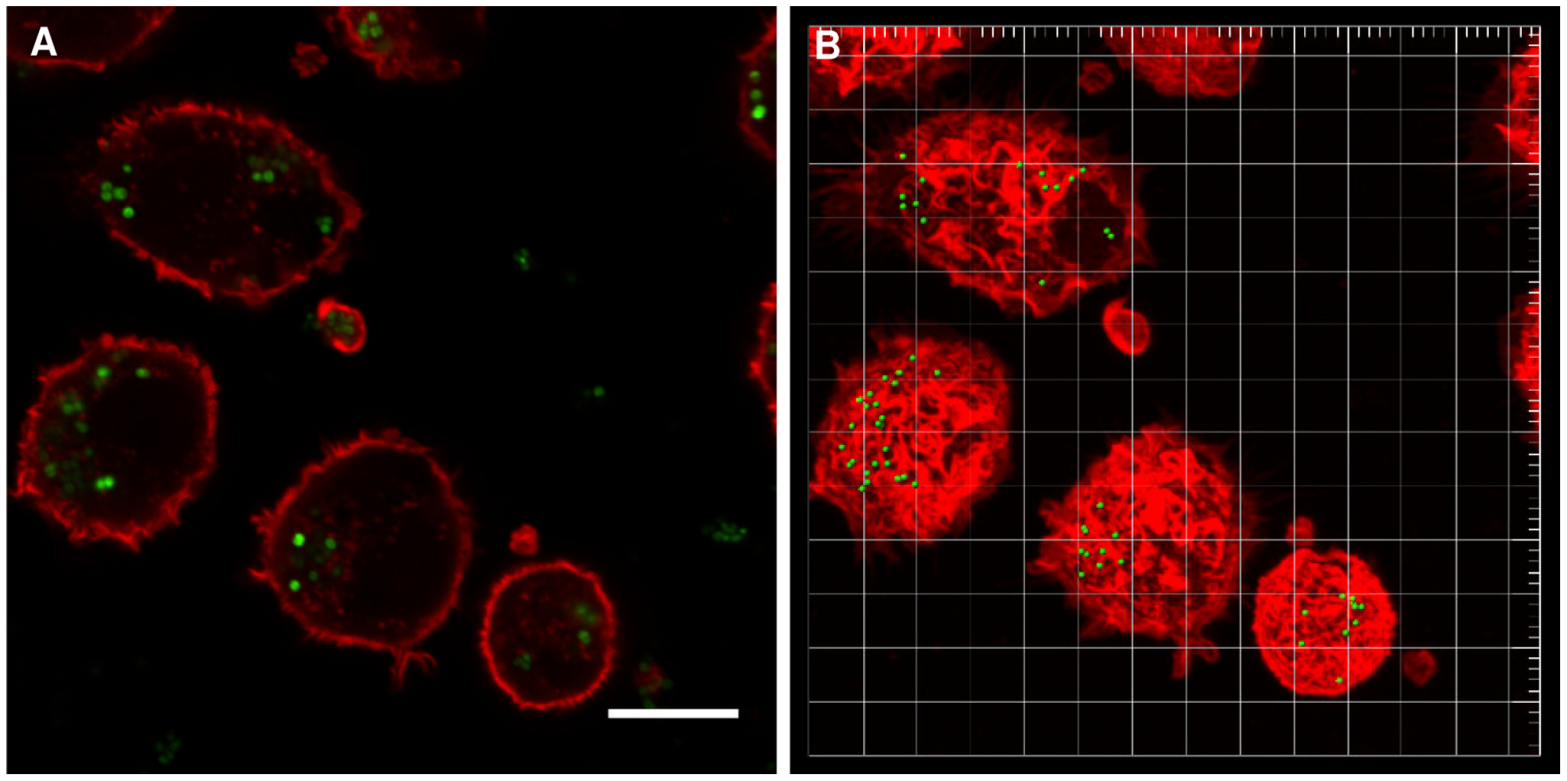
Direct quantification of *S. aureus* ATCC12600^GFP^ inside phagocytes using CLSM. (A) CLSM snapshot of *S. aureus* ATCC 12600^GFP^ (modified to express GFP) inside J774A.1 macrophages stained with TRITC-phalloidin and (B) reconstruction of 3D image from CLSM sections using Bitplane’s Imaris software. Note that bacteria appear green fluorescent, while the cell wall of the phagocytes is red. Bar denotes 20 µm.

**Figure 2:**
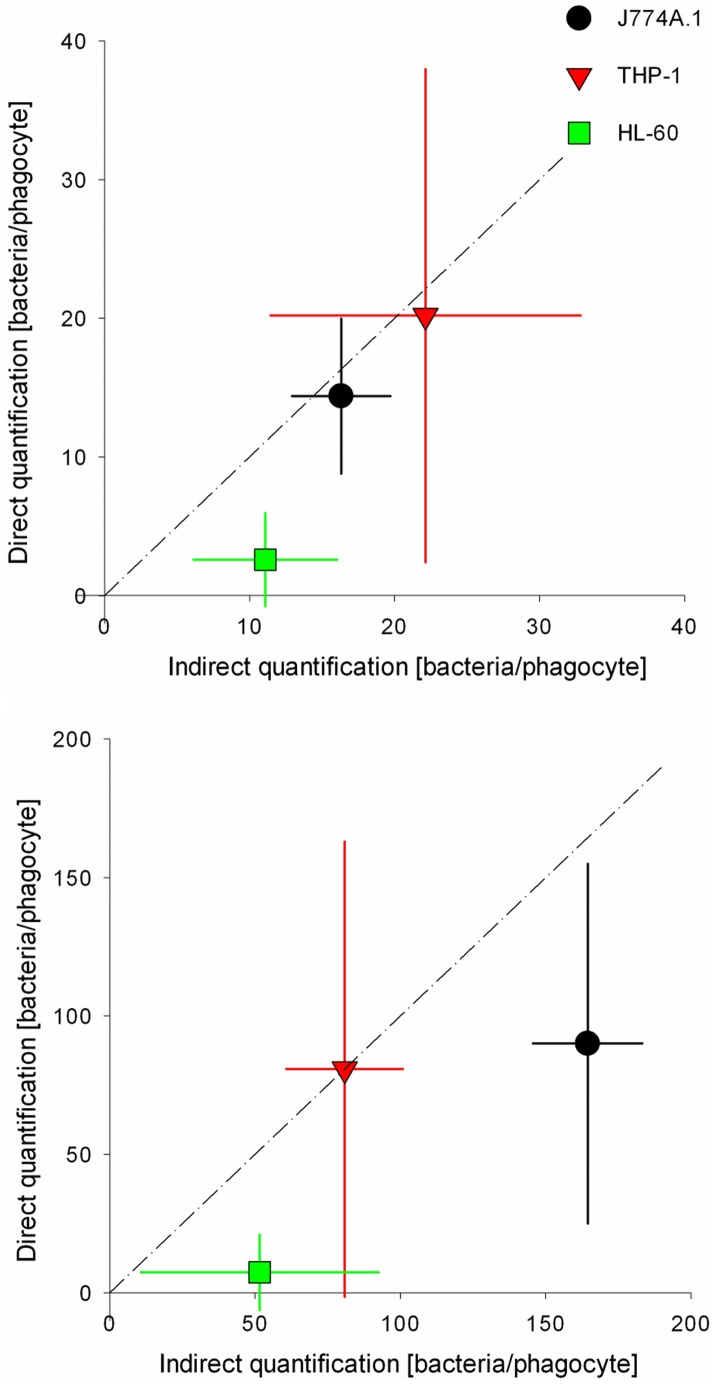
Comparison of direct *versus* indirect quantification of staphylococcal phagocytosis. (A) staphylococcal adhesion for 1 h to a density of approximately 1.2×10^6^ bacteria/cm^2^ and (B) staphylococcal adhesion for 3.5 h to a density of approximately 8×10^6^ bacteria/cm^2^. The line indicates complete correspondence between both methods. Error bars represent the standard deviations over three replicates, with separately cultured bacteria and phagocytes.

As an example of the direct method, Figure 1 shows a CLSM snapshot and 3D image reconstruction of *S. aureus* ATCC 12600^GFP^ inside a murine J774A.1 macrophage (see supplementary information, Video S2). Single bacteria can be clearly seen, allowing enumeration of the number of bacteria inside a single phagocytic cell. A quantitative comparison of the number of fluorescent staphylococci inside a single phagocytic cell obtained using the direct and indirect method is presented in Figure 2. Data for the three phagocytic cell lines fall close to the line of identity. The most important difference between the direct and the indirect method appears in the region where the numbers of bacteria removed by phagocytosis is low. In cases where the direct method yields absence of phagocytized bacteria, the indirect method yields 10 to 50 bacteria per phagocyte. This points to a clear advantage of the indirect method, encompassing a much larger number of phagocytic cells than can be achieved with the direct method. In addition, through the indirect method, all bacteria present on the surface (dead or live) are being quantified, while that is not possible with the direct quantification that only focuses on metabolically active bacteria. Fewer bacteria were engulfed by phagocytic cells when the number of adhering staphylococci was low.

**Figure 3:**
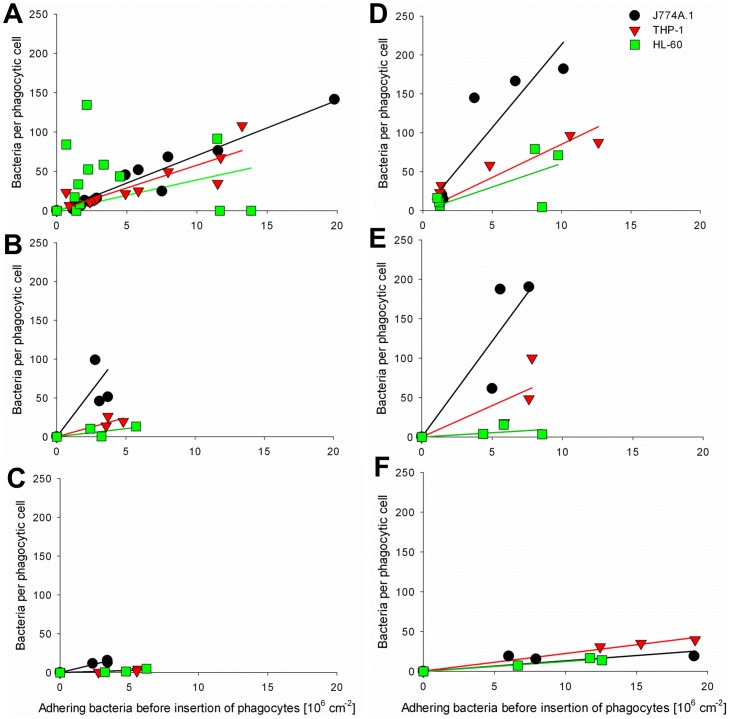
*Staphylococci* per phagocyte after 2 h *versus* the number of adhering bacteria initially present. (A) *S. epidermidis* 3399 after 1 h, 3.5 h, 14 h and 24 h of bacterial growth, (B) *S. epidermidis* 7391 after 3.5 h bacterial growth, (C) *S. epidermidis* 1457 after 3.5 h bacterial growth, (D) *S. aureus* ATCC 12600^GFP^ after 1 h and 3.5 h of bacterial growth, and (E) *S. aureus* 7323 after 3.5 h bacterial growth, (F) *S. aureus* LAC after 3.5 h bacterial growth. Solid lines indicate the best-fit to a linear function passing through origin.

The dependence of the number of bacteria internalized on the number of bacteria adhering to the substratum surface indicates that in order to obtain a single parameter for phagocytosis of adhering bacteria, results have to be normalized with respect to the number of adhering bacteria before insertion of phagocytes (see also supplementary information, Figure S1 for more detail). Figure 3 reveals that the number of staphylococci phagocytized depends linearly on the number of adhering bacteria, regardless of the bacterial strain or phagocytic cell line involved. According to the slope of the lines in Figure 3 (see supplementary information, Table S1 for R^2^ values), and accounting for the fact that phagocytosis was pursued for 120 min, a “phagocytosis rate” can be calculated, as summarized in Table 1 for all staphylococcal strains and the three phagocytic cell lines. Phagocytosis rates depended very much on the combination of the staphylococcal strain and phagocytic cell line involved. In general however, the murine macrophage cell line (J774A.1) was more efficient in internalizing adhering staphylococci than the two human phagocytic cell lines.

**Table 1:** Phagocytosis rates for six staphylococcal strains by different phagocytic cell lines (cm^2^/min).

Bacterial strain	J774A.1 (murine) (×10^−8^)	THP-1 (human) (×10^−8^)	HL-60 (human) (×10^−8^)
***S. epidermidis*** **3399**	5.8±0.8	4.8±1.1	3.3±4.0
***S. epidermidis*** **7391**	15.5±8.8	4.1±1.3	1.7±1.1
***S. epidermidis*** **1457**	3.6±0.5	0.4±0.2	0.4±0.3
***S. aureus*** **ATCC 12600^GPF^**	17.8±4.3	6.9±1.4	4.9±2.6
***S. aureus*** **7323**	20.5±7.1	6.8±3.8	0.9±0.9
***S. aureus*** **LAC**	1.1±0.6	1.9±0.2	1.0±0.2

±signs indicate the range of uncertainty based on a confidence level ≥95%.

### Surface Thermodynamic Analysis of Phagocytosis Rates

In order to determine whether differences in phagocytosis rates of the different staphylococcal strains by the three phagocytic cell lines could be explained on a surface thermodynamic basis, contact angles were measured on lawns of phagocytes and bacteria with water, formamide, methylene iodide and α-bromonaphthalene (Table 2). All cells in this study had a water contact angle lower than 65 degrees, indicating hydrophilic behavior [20]. Water contact angles on all phagocytic cell lines were comparable between 46 and 51 degrees, while water contact angles among the different staphylococcal strains varies over a wider range from 23 to 59 degrees. Contact angles of all four liquids were employed to calculate the surface free energy parameters and components of the cell surfaces. In all cases, the acid-base surface free energy component was comprised of a large electron-donating parameter, in combination with a small electron-accepting one. The larger hydrophilicities of *S. epidermidis* 3399, *S. epidermidis* 7391, *S. aureus* 7323 and *S. aureus* LAC become evident in a significantly larger electron-donating surface free energy parameter than found for the other strains (p<0.001). The murine macrophage cell line (J774A.1) had the smallest electron-donating surface free energy parameter among the phagocytic cell lines. The acid-base surface free energy component follows directly from the electron-donating and electron-accepting parameters and on average is significantly (p<0.001) higher for the phagocytic cell lines than for the staphylococcal strains. The Lifshitz-Van der Waals component follows from the contact angles with the a-polar liquids (methylene iodide and α-bromonaphthalene) and shows relatively little variation across phagocytic cell lines and staphylococcal strains.

**Table 2:** Contact angles of water (θ_w_), formamide (θ_f_), methyleniodide (θ_m_) and α-bromonaphthalene (θ_b_) measured on lawns of the staphylococcal strains and phagocytic cell lines involved in this study(in degrees).

Cell type	θ_w_	θ_f_	θ_m_	θ_b_	γ^-^	γ^+^	γ^AB^	γ^LW^	γ^tot^
*S. epidermidis* 3399	28±2c,d,g–i	30±4c,f–h	49±1b,g–i	25±4d,g,i	51±4d,g–i	0.7±0.3c,f–i	11±3c,f,g–i	37±1^g–i^	49±2c,f
*S. epidermidis* 7391	33±2^c–e,g–i^	36±3c,e–i	56±2a,c–f,h,i	19±3d,g,i	48±2d,g–i	0.5±0.1c,f–i	9±0.4c,f,g–i	36±0.2^g–i^	46±1c,f,h,i
*S. epidermidis* 1457	46±3a,b,d–f	57±3a,b,d–i	46±3a,b,d–i	18±3d,g,i	53±1d,g–i	0±0,0a,b,d,e,g–i	0±0.0a,b,d,e,g–i	39±2.6^g–i^	39±1a,b,d,e,g–i
*S. aureus* ATCC12600^GFP^	59±2^a–c,e–i^	40±5a,c,e,g–i	48±1b,g–i	36±4^a–c,e–h^	18±2^a–c,^ e,f	1.4±0.7c,f–i	10±2c,f,g–i	36±1^g–i^	45±2c,f,h,i
*S. aureus* 7323	23±2^b–d,f–i^	27±3b,c,e,f,g,h	47±1b,g–i	26±2d,g	53±2d,g–i	0.8±0.2c,f–i	12±2c,f,g–i	38±0.1^g–i^	50±2c,f
*S. aureus* LAC	34±4^c–e,g–i^	47±5^a–c,^ e,g–i	50±4b,g–i	25±3d,g,i	59±6d,g–i	0.01±0.02a,b,d,e,g–i	0.8±1a,b,d,e,g–i	37±0.6^g–i^	38±1a,b,d,e,g–i
J774A.1 (murine)	51±2a,b.d–f	17±5^a–f^	60±1a,c–f,h,i	52±2^a–f,^ h,i	15±1^a–c,^ e,f	7.7±0.4^a–f^	22±1^a–f^	29±1^a–f^	51±1c,f
THP-1 (human)	46±5a,b,d–f	13±2^a–f,^ i	84±2^a–g,^ i	26±2d,g,i	18±2^a–c,^ e,f	7.7±0.3^a–f^	24±2^a–f^	28±1^a–f^	52±1b,c,d,f
HL-60 (human)	46±2a,b,d–f	25±1^b–d,^ f,h,	67±2^a–h^	33±3^a–c,^ f,g	23±4^a–c,^ e,f	5.3±1.9^a–f^	22±2^a–f^	30±2^a–f^	52±1b,c,d,f

Surface free energy parameters and components are derived from the measured contact angles, yielding an electron-donating (γ^-^) and electron-accepting (γ^+^) parameter for the acid-base component (γ^AB^), a Lifshitz-Van der Waals component (γ^LW^) and the total surface free energy (γ^tot^) (mJ/m^2^). ± signs indicate standard deviations over three separately prepared lawns, taking three measurements of different liquid droplets on each lawn.

a significantly different from *S. epidermidis* 3399;

b significantly different from *S. epidermidis* 7391;

c significantly different from *S. epidermidis* 1457;

d significantly different from *S. aureus* ATCC12600^GFP^;

e significantly different from *S. aureus* 7323;

f significantly different from *S. aureus* LAC.

g significantly different from J774A.1;

h significantly different from HP-1;

i significantly different from HL-60. All significance levels were indicated at p<0.001.

**Figure 4:**
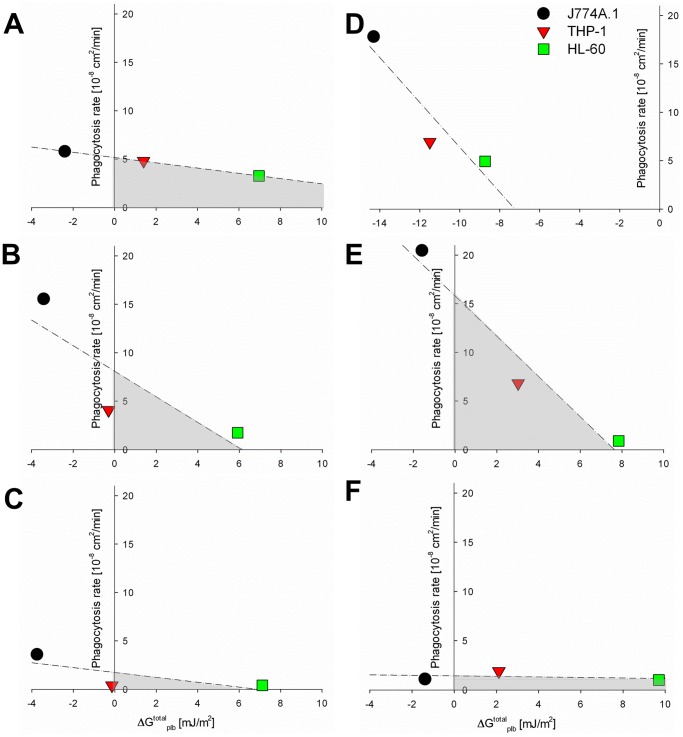
Phagocytosis rate *versus* the interfacial free energy of adhesion. (A) S. epidermidis 3399, (B) S. epidermidis 7391, (C) S. epidermidis 1457, (D) *S. aureus* ATCC 12600^GFP^, (E) *S. aureus* 7323, and (F) *S. aureus* LAC. Note that phagocytosis rates increase when the interfacial free energy of adhesion becomes more favorable (more negative), but phagocytosis is not ruled out by unfavorable surface thermodynamic conditions (shaded regions). Dashed lines indicate the best-fit to a linear function through the data.

The surface free energies summarized in Table 2 can be employed to calculate the Lifshitz-Van der Waals (ΔG^LW^_plb_) and acid-base (ΔG^AB^_plb_) contributions to the interfacial free energy of adhesion between phagocytic cells and staphylococci (Table 3). Long-range attraction between phagocytes and staphylococci is always thermodynamically favorable, as indicated by negative ΔG^LW^_plb_ values, but differences appear in short-range interactions. Acid-base interactions are favorable toward adhesion of the *S. aureus* ATCC12600^GFP^ strain to all phagocytic cells, while ΔG^AB^_plb_ values were far less negative (less favorable for adhesion to occur) or even positive for the human phagocytic cell lines in case of the other strains. Figure 4 shows the relation between interfacial free energy of adhesion and the rate of phagocytosis for the six staphylococcal strains by the different phagocytic cell lines. From the absence of positive slopes in Figure 4, it can be concluded that phagocytosis follows surface thermodynamic principles and occurs more readily when the interfacial free energies of adhesion are more negative. However, absence of favorable surface thermodynamic conditions does not rule out phagocytosis (see Figure 4, shaded regions).

**Table 3:** Lifshitz-Van der Waals and acid-base components of interfacial free energy of adhesion (ΔG^LW^_plb_ and ΔG^AB^_plb_, respectively) between bacteria and phagocytes, calculated from measured contact angles with liquids, as presented in Table 2 (mJ/m^2^).

Bacterial strains		Phagocytes		
		J774A.1 (murine)	THP-1 (human)	HL-60 (human)
***S. epidermidis*** ** 3399**	ΔG^LW^_plb_	−2.1±0.1	−2.0±0.4	−2.3±0.5
	ΔG^AB^_plb_	−0.3±1.8	3.4±2.4	9.3±5.2
	**ΔG^total^_plb_**	**−2.4±1.8** c,i–l,n,o,r	**1.4±4.9^ j–l^**	**6.9±4.9** a,d,j–m,p
***S. epidermidis*** ** 7391**	ΔG^LW^_plb_	−2.0±0.1	−1.8±0.3	−2.2±0.5
	ΔG^AB^_plb_	−1.4±1.1	1.5±2.9	8.1±4.6
	**ΔG^total^_plb_**	**−3.4±1.2** c,f,i–k,n,o,q,r	**−0.3±3.2** j,k,o,r	**5.9±4.2** d,g,j–m,p
***S. epidermidis*** ** 1457**	ΔG^LW^_plb_	−2.4±0.1	−2.1±0.3	−2.6±0.4
	ΔG^AB^_plb_	−1.4±1.1	1.9±2.3	9.7±5.0
	**ΔG^total^_plb_**	**−3.7±1.1** f,i–o,q,r	**−0.1±2.6^j–l,^** o,r	**7.1±4.7** a,d,g,j–l,p
***S. aureus*** ** ATCC12600^GFP^**	ΔG^LW^_plb_	−1.9±0.1	−1.7±0.2	−2.1±0.3
	ΔG^AB^_plb_	−12.6±0.9	−9.9±2.4	−6.6±3.1
	**ΔG^total^_plb_**	**−14.3±1.0^a–i,l–r^**	**−11.6±2.6^a–h,I–r^**	**−8.7±3.2^a–j,m–r^**
***S. aureus*** ** 7323**	ΔG^LW^_plb_	−2.2±0.1	−1.8±0.3	−2.4±0.4
	ΔG^AB^_plb_	0.6±0.7	4.8±1.7	10.3±5.4
	**ΔG^total^_plb_**	**−1.6±0.7** c,f,g,i–l,n,o,q,r	**3.0±2.1** a,d.g,j–m,p	**7.8±4.5** a,d,e,g,h,j–m,p
***S. aureus*** ** LAC**	ΔG^LW^_plb_	−2.1±0.1	−1.9±0.3	−2.3±0.4
	ΔG^AB^_plb_	0.7±1.7	4.0±2.6	12±5.5
	**ΔG^total^_plb_**	**−1.4±1.7** c,f.i–l,n,o,r	**2.1±2.8** d,g,j–m	**9.8±5.2** a,d,e,g,h,j–m,p

a significantly different from *S. epidermidis* 3399 adhering to J774A.1;

b significantly different from *S. epidermidis* 3399 adhering to THP-1;

c significantly different from *S. epidermidis* 3399 adhering to HL-60;

d significantly different from *S. epidermidis* 7391 adhering to J774A.1;

e significantly different from *S. epidermidis* 7391 adhering to THP-1;

f significantly different from *S. epidermidis* 7391 adhering to HL-60;

g significantly different from *S. epidermidis* 1457 adhering to J774A.1;

h significantly different from *S. epidermidis* 1457 adhering to THP-1;

i significantly different from *S. epidermidis* 1457 adhering to HL-60;

j significantly different from *S. aureus* ATCC12600^GFP^ adhering to J774A.1;

k significantly different from *S. aureus* ATCC12600^GFP^ adhering to THP-1;

l significantly different from *S. aureus* ATCC12600^GFP^ adhering to HL-60;

m significantly different from *S.aureus* 7323 adhering to J774A.1;

n significantly different from *S.aureus* 7323 adhering THP-1;

o significantly different from *S.aureus* 7323 adhering to HL-60;

p significantly different from *S. aureus* LAC adhering to J774A.1;

q significantly different from *S. aureus* LAC adhering to THP-1;

r significantly different from *S. aureus* LAC adhering to HL-60. All significance levels were indicated at p<0.001.

## Discussion

Biomaterial-associated-infections have been the number one cause of failure of biomaterials implants and devices for decades now with disastrous consequences for those afflicted [2]–[4]. Biomaterial-associated-infections are highly recalcitrant, partly because the immune system is impaired around a biomaterial implant or device [2], [3], [5]. The details of the interaction between adhering pathogens and phagocytes are not well understood, but it is evident that bacteria have developed strategies to evade the response of phagocytic cells [3], [27]. Here we present a method by which phagocytosis of bacteria adhering to a surface can be quantified in a parallel plate flow chamber, and expressed as a phagocytosis rate, accounting for the number of adhering bacteria initially present and the duration of phagocytosis. Furthermore we show that a thermodynamic approach can be applied to explain the interaction between phagocytic cells and adhering staphylococci at the level of a specific strain. No ubiquitously valid relation between phagocytosis rate and interfacial free energies of adhesion was found however. This shows that there are also other mechanisms involved in phagocytosis and hydrophobic interactions, as expressed in the interfacial free energies of adhesion, do not operate on their own.

Bacterial phagocytosis was quantified by a direct and an indirect method. Methods were compared for a GFP producing *S. aureus* strain and showed comparable numbers of staphylococci phagocytized per phagocyte (Figure 2). This is despite the fact, that the indirect method comprised both the removal of dead and live staphylococci from the surface, whereas the fluorescence based direct method only included metabolically active bacteria. The advantage of the indirect quantification is that it is easy, relatively fast and allows working with non-fluorescent clinical isolates, while furthermore interactions between phagocytes and bacteria can be followed in real-time. However, when the number of adhering bacteria becomes too high and 3D biofilms start to develop, microscopic segmentation of single bacteria by phase contrast microscopy becomes difficult. Good segmentation of single bacteria inside phagocytic cells by 3D reconstruction may remain possible, however, but this cannot be done in real-time. Therefore, we considered the indirect method to be more versatile.

Phagocytosis rates increase when the interfacial free energy of adhesion ΔG_plb_^total^ between phagocytes and staphylococci becomes more negative (Figure 4), which is in line with a surface thermodynamic analysis based on measured contact angles with liquids on macroscopic lawns of staphylococci and phagocytic cells. Like in nearly all biological interactions [4], Lifshitz-Van der Waals forces mediate the long-range attraction between phagocytes and bacteria, but short-range acid-base interactions dictate the overall macroscopic adhesion and therewith whether favorable thermodynamic conditions exist for phagocytosis to occur. Although it was known that phagocytosis of pathogens in planktonic state could be explained on a surface thermodynamic basis [21], [22], [24], [28], our study shows that this also holds for adhering pathogens. Importantly, this confirms the role of direct physical adhesion between phagocytes and pathogens in effective phagocytosis. *S. aureus* ATCC12600^GFP^ interaction was thermodynamically favorable to all phagocyte cell lines included, while all other bacterial strains included only possessed thermodynamically favorable conditions for adhesion to the murine cell line (J774A.1). *S. epidermidis* 7391 and *S. epidermidis* 1457 also revealed slightly favorable thermodynamic conditions for adhesion to THP-1 (ΔG_plb_^total^ = −0.3 mJ/m^2^ and ΔG_plb_^total^ = −0.1 mJ/m^2^, respectively).

Previous studies have focused on chemo-attraction as the main driving force for bacterial phagocytosis [5], [7], [29], [30], and chemo-attractants may well explain phagocytosis of adhering bacteria by human phagocytic cell lines despite unfavorable thermodynamic conditions for direct physical interaction (see Figure 4 shaded regions). Bacterial cell wall peptidoglycan and lipoteichoic acids are known to be powerful in activating phagocytes [2], [31], [32]. For example, *S. aureus* 7323 the most hydrophilic strain, with only a small favorable thermodynamic condition for the murine cell line, showed high phagocytosis rates for the murine and human THP-1 cell lines, despite the thermodynamical unfavorable condition for the latter. In contrast, it has been shown that bacteria can keep phagocytes in an inactive state by adhesins, such as polysaccharide intercellular adhesins (PIA), accumulation-associated protein and extracellular matrix binding protein [2], [3], [33], [34]. The production of PIA by *S. epidermidis* 1457 in a biofilm mode of growth for instance [3] kept this bacterium “under the radar” of immune cells, which is in line with our results revealing one of the smallest phagocytosis rates of all phagocytic cells for this staphylococcal strain. In addition, also *S. aureus* LAC, a highly virulent MRSA strain [2], [33], revealed small phagocytosis rates, which can be due to the secretion of phenol-soluble modulins molecules enhancing the ability of this pathogen to evade immune cell action [2], [33]. Hence, phagocytosis must be regarded as an interplay between physico-chemical attraction and influences of chemo-attractants.

Highly important for future studies, our study is the first to demonstrate quantitative differences in phagocytosis rates of adhering staphylococci by different phagocytic cell lines. The murine macrophage cell line (J774A.1) showed higher phagocytosis rates than both human cell lines (see Table 1), likely because the human cell lines are monocytic (THP-1) and promyelocytic (HL-60) cell lines, accustomed to growth in suspension rather than on a surface. These cell lines therefore first need to differentiate into macrophages or neutrophils during an experiment and develop the ability to adhere in order to effectively phagocytize bacteria, which takes time. In order to demonstrate this, we differentiated HL-60 promyelocytic cells into neutrophils by exposing them to phorbol 12-myristate 13-acetate for 24 h prior to experiments [34]. Differentiated cells indeed showed an increase in phagocytosis rate of adhering *S. epidermidis* 3399 from 3.3×10^−8^ to 7.2×10^−8^ cm^2^/min. Unfortunately, differentiated cells are extremely difficult to harvest viably due to their strong adhesion to culture flasks, which impedes routine experiments with differentiated cells. Accordingly, different phagocytic cell lines commonly used in *in vitro* studies may have different phenotypes and release different cytokine profiles under specific conditions, in which the type of biomaterial plays an important role as well [35], [36].

This study demonstrates that phagocytosis critically depend on the combination of the phagocytic cell line and the bacterial strain involved. Differences in phagocytosis rates at the level of a specific staphylococcal strain could be largely explained on a surface thermodynamic basis using measured contact angles of liquids on macroscopic lawns of staphylococci and phagocytes. Differences were found between murine and human phagocytic cell lines. Therewith, the present findings will aid to put results of future studies on biomaterial-associated-infections in murine infection models in better perspective with respect to human ones. Future research should focus on identifying the differences between the immune cells of murine models *versus* immune cells of human host.

## Materials and Methods

### Bacterial Culture Conditions and Harvesting

Six staphylococcal strains were used in this study: *S. epidermidis* 3399 (isolated from human skin), *S. epidermidis* 7391 (clinical isolate from an infected hip arthroplasty), *S. epidermidis* 1457 (isolated from an infected central venous catheter), *S. aureus* 7323 (clinical isolate from an infected joint arthroplasty), *S. aureus* LAC (also named USA300, a methicillin resistant *Staphylococcus aureus* isolate) and *S. aureus* ATCC 12600 (isolated from pleural fluid), modified to express Green Fluorescent Protein (GFP in pMV158 plasmid) [26], and noted as *S. aureus* ATCC 12600^GFP^. All bacteria were first grown aerobically overnight at 37°C on blood agar plates from a frozen stock, except *S. aureus* ATCC 12600^GFP^ that was grown using tryptone soya broth (TSB; OXOID, Basingstoke, UK) agar plates with 1% tetracyclin (Sigma-Aldrich, Steinheim, Germany). For the experiments with *S. aureus* ATCC 12600^GFP^, TSB was always supplemented with 1% tetracyclin to select bacteria expressing GFP. One colony of each bacterial strain was inoculated separately in 10 ml TSB and cultured for 24 h at 37°C. These cultures were then used to inoculate a second culture in 200 ml TSB, grown for 17 h at 37°C. Bacteria were harvested by centrifugation (5 min at 5000×g at 10°C) and washed twice with sterile phosphate-buffered saline (PBS, 10 mM potassium phosphate, 0.15 M NaCl, pH 7.0). Subsequently, the bacteria harvested were sonicated on ice (3×10 s) in PBS to break bacterial aggregates. Bacteria were resuspended in sterile PBS to a concentration of 3×10^8^ bacteria/ml, as determined in a Bürker-Türk counting chamber.

### Culture Conditions and Harvesting of Phagocytes

Three phagocytic cell lines, all obtained from LGC (Wesel, Germany) were used: one murine macrophage cell line (J774A.1) and two human cell lines (monocytic THP-1 and promyelocytic HL-60). All phagocytic cell lines were grown in tissue culture polystyrene flasks (Greiner Bio-One, Frickenhausen, Germany), and routinely cultured in Dulbecco’s Modified Eagle’s Medium supplemented with 4.5 g/l D-glucose, pyruvate and 10% fetal bovine serum (referred in this article as DMEM-HG +10% FBS; reagents obtained from Sigma-Aldrich, Steinheim, Germany). Phagocytes were maintained at 37°C in a humidified atmosphere with 5% CO_2_. The human cell lines were passaged each two days, and the murine cell line was passaged at 70–80% confluence by scraping. Cells were harvested by centrifugation (5 min at 150×g) in DMEM-HG +10% FBS previous to experiments. The harvested cells were counted using a Bürker-Türk hemocytometer and subsequently diluted to a concentration of 6×10^5^ cells/ml.

### Bacterial Adhesion and Phagocytosis

Staphylococci were allowed to adhere to glass slides in a parallel plate flow chamber (175×17×0.75 mm^3^), equipped with heating elements and kept at 37°C throughout the experiments. Prior to each experiment, all tubes and the flow chamber were filled with sterile PBS, taking care to remove all air bubbles from the system. PBS was perfused through the system at a shear rate of 11 s^−1^. Then, a bacterial suspension was perfused through the chamber at the same shear rate and phase-contrast images were obtained. Bacteria were allowed to adhere to the glass surface for 30 min while enumerating their number (bacteria/cm^2^), as assessed by an automated counting algorithm. Flow was switched to sterile PBS to remove non-adhering bacteria from the tubes and chamber. After 30 min, PBS was replaced by TSB, which was perfused for different periods of time to allow bacterial growth (see supplementary information, Figure S2 for an example) but restricted to growth of bacterial monolayers to facilitate bacterial enumeration by phase contrast microscopy (the “indirect” method). While other strains already formed 3D structures after 3.5 h of growth in TSB, *S. epidermidis* 3399 grew much slower and monolayers were still obtained after 24 h of growth in TSB. Subsequently, DMEM-HG +10% FBS was perfused for 10 min to remove non-adhering bacteria. Next, medium was replaced by a suspension of phagocytic cells (6×10^5^ cells/ml) and the flow was stopped for 2 h to allow the phagocytes to settle and phagocytosis to occur (see supplementary information, Video S1). All experiments were done in triplicate with three independent bacterial and phagocytic cell cultures.

Two methods were used to quantify the number of phagocytized bacteria. One method was based on phase contrast imaging (indirect quantification) while the other method was based on confocal laser scanning microscopy (CLSM, direct quantification).

Indirect quantification: Bacterial growth and phagocytosis by phagocytes on the glass surface was followed in real-time with a CCD camera (Basler AG, Germany) mounted on a phase-contrast microscope Olympus BH-2 (Olympus, Germany) with a 40× objective. After bacterial growth in the parallel plate flow chamber, different phagocytes were inserted in the flow chamber and phagocytosis was followed in real-time for 2 h in the absence of flow. In order to verify that no bacteria detached during these conditions, similar experiments were done in absence of phagocytes (see supplementary information, Table S2). The number of bacteria phagocytized by a single phagocytic cell was indirectly determined by assessing the number of adhering bacteria to glass per unit area, using proprietary software based on the Matlab Image processing Toolkit (The MathWorks, Natick, MA, USA), and subtracting the number of bacteria that had remained adhering from the control enumerations (see also supplementary information, Figure S1 and Tables S2 and S3).

Direct quantification: Direct quantification was only done with *S. aureus* ATCC12600^GFP^. Initially, experiments were conducted as described above for the indirect method, but after 2 h of phagocytosis, the glass surfaces were removed from the flow chamber and prepared for CLSM. To this end, glass slides with adhering staphylococci and phagocytes were fixed with 3.7% formaldehyde in cytoskeleton stabilization buffer (CS; 0.1 M Pipes, 1 mM EGTA, 4% (w/v) polyethylene glycol 8000, pH 6.9) for 15 min. Subsequently, glass slides were incubated in 0.6% Triton X-100 for 3 min, rinsed with PBS and stained for 30 min with PBS containing 2 µg/ml of Tetramethyl Rhodamine Isothiocyanate (TRITC)-phalloidin, followed by washing with PBS. TRITC-phalloidin was used to stain the phagocytes actin cytoskeleton. Image stacks (1024×1024 pixels) with optical slices of less than 1 µm were taken with CLSM (Leica DMRXE with confocal TCS SP2 unit, Mannheim, Germany) using an HCX PL APO 63/1.32 NA oil immersion lens (pinhole was set at its optimal value of 1.0 according to the manufacturer specifications), on nine different locations of the sample (see supplementary information, Video S2 for an example of a confocal 3D stack of images). CLSM images were reconstructed and analyzed in 3D with Imaris software (Bitplane AG, Zurich, Switzerland) and the number of *S. aureus* ATCC 12600^GFP^ inside each phagocytic cell was evaluated.

### Contact Angle Measurements and Surface Thermodynamics Analysis

Cell surface free energies of the different bacterial strains and phagocytic cell lines were determined by measuring advancing-type contact angles at room temperature (25°C) using the sessile drop technique with a home-made contour monitor. Bacteria or phagocytes were layered onto a 0.45 µm pore-size HA membrane filter (Millipore Corporation, Bedford, MA, USA) using negative pressure. The filters containing the cells were placed on a metal disc and allowed to air dry until so-called “plateau” water contact angles could be measured, representing a state of drying in which loose water was removed while cells remain in a physiological, hydrated state. To this end, water contact angles were measured on lawns of each cell type to determine the plateau level of drying, which usually amounted between 60 and 180 min. Three independent cultures were used for each bacterial strain and phagocytic cell line, and three lawns were prepared out of each culture.

To allow surface thermodynamic analysis, contact angles were measured with four different liquids (water, formamide, methylene iodide and α-bromonaphthalene) [6], [21], [24]. Due to the different polarities of these liquids, their contact angles enable calculation of the cell surface free energy (γ^tot^), its LW (γ^LW^) and AB (γ^AB^) components and the electron-donating and accepting parameters. The bacterial and phagocyte surface free energies were subsequently employed to calculate the free energy of adhesion (ΔG_plb_) between the bacterial (b) and phagocyte (p) cell-surface in an aqueous suspension (l) and its LW (ΔG_plb_^LW^) and AB (ΔG_plb_^AB^) components, according to
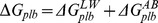
(1)where
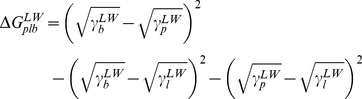
(2)and



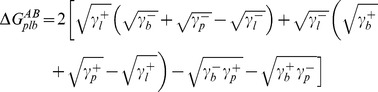
(3)According to surface thermodynamics, adhesive interactions will be favorable if ΔG_plb_ <0 [4], [6].

### Statistics

Data are presented as a mean with standard deviation. ANOVA was applied followed by a Tukey’s HSD post-hoc test to demonstrate statistically significant differences and p-values smaller than 0.001 were considered significant.

## Supporting Information

Figure S1 **Phase contrast images of adhering bacteria after 14 h of growth and 2 h interaction between bacteria and murine macrophages (J77A.1).** A) *S. epidermidis* 3399 after 14 h of growth and 2 h in contact with macrophage medium in absence of macrophages (control), B) *S. epidermidis* 3399 after 14 h of growth and 2 h interaction with J774A.1, C) Fig. S1A processed with proprietary software, placing a green dot on top of each bacterium recognized by the software, and D) Fig. S1B processed with proprietary software, placing a green dot on top of each bacterium outside J774A.1. Processed images were used to access the number of adhering bacteria to glass per unit area. One field of view in the above series of images covers a surface area of 6.75×10^−4^ cm^−2^, from which by conversion the number of adhering bacteria/cm^−2^ can be easily calculated for images taken after different growth times (see Figure S2), as summarized in Table S2. The difference between the number of adhering bacteria in the presence and in the absence of phagocytes provides with the total number of phagocytized bacteria. Control experiments in absence of phagocytes allow to account for any bacterial growth occurring during 2 h interaction with phagocytes. Since the total number of bacteria phagocytized depends on the number of phagocytic cells present, we also determined the number of phagocytes adhering per unit area, as described above for the adhering staphylococci (see summary in Table S3). Once knowing the number of phagocytes and phagocytized bacteria per unit area, the numbers of bacteria phagocytized within one phagocytic cell follows (see further Video S1).
(TIF)

Figure S2 **Phase-contrast images of the growth of adhering** ***Staphylococci epidermidis*** **3399 for different periods of time.** Staphylococcal adhesion and growth on a glass surface at 37°C and under constant shear (11 s^−1^). Scale bar represents 40 µm.
(TIF)

Figure S3 **Colony formation units (CFU) per ml of the bacteria grown in solution or detached from the surface after 2 h interaction, in the presence and absence of murine macrophages (J774A.1).** No statistical differences were found between the number of bacteria found in solution in the absence and presence of phagocytes. Experiments were done in tissue culture polystyrene well plates (static conditions), where 1 ml of bacterial suspension (1×10^6^ bacteria/ml) was grown for 1 h in tryptone soya broth (TSB) at 37°C. Subsequently non-adhering bacteria were removed by washing three times with DMEM-HG+10%FBS and 1 ml of macrophage suspension (6×10^4^ bacteria/ml) or medium without macrophages (control) was added and incubated for 2 h. After 2 h, the medium in each well, with bacteria-macrophages or bacteria-medium, was diluted 1000× and 100 µl was plated on TSB agar plates. Bacterial colonies were quantified after 24 h incubation to identify the number of bacteria that detach during interaction with and without macrophages. Experiments were done in triplicate.
(TIF)

Table S1 **R^2^ values of a linear fit through the data describing the number of staphylococci internalized per phagocyte as a function of the number of initially adhering staphylococci.** Note, that the linear function was mathematically forced to pass through the origin, i.e. zero staphylococci internalized when the number of initially adhering staphylococci is zero (see Figure 3).
(DOC)

Table S2 **Number of staphylococci (10^6^ bacteria/cm^2^) adhering to glass after growth for different periods of time, in absence (control) and presence of phagocytes.** Data are presented prior to (interaction time 0 h) and after interaction with phagocytes (interaction time 2 h).
(DOC)

Table S3 **The number of phagocytes (10^4^ phagocytes/cm^2^) present in the individual experiments.**
(DOC)

Video S1 **Indirect quantification of** ***S. aureus*** **ATCC12600^GFP^ phagocytosis by J774A.1.**
(WMV)

Video S2 **CLSM z-stack video for J774A.1 interaction with** ***S. aureus*** **ATCC12600^GFP^.**
(WMV)
