# Supplementary figures and images for: Phagocytosis of Bacteria Adhering to a Biomaterial Surface in a Surface Thermodynamic Perspective
Source: PLoS One. 2013 Jul 19;8(7):e70046. doi: 10.1371/journal.pone.0070046 (PMC3716708; doi:10.1371/journal.pone.0070046)

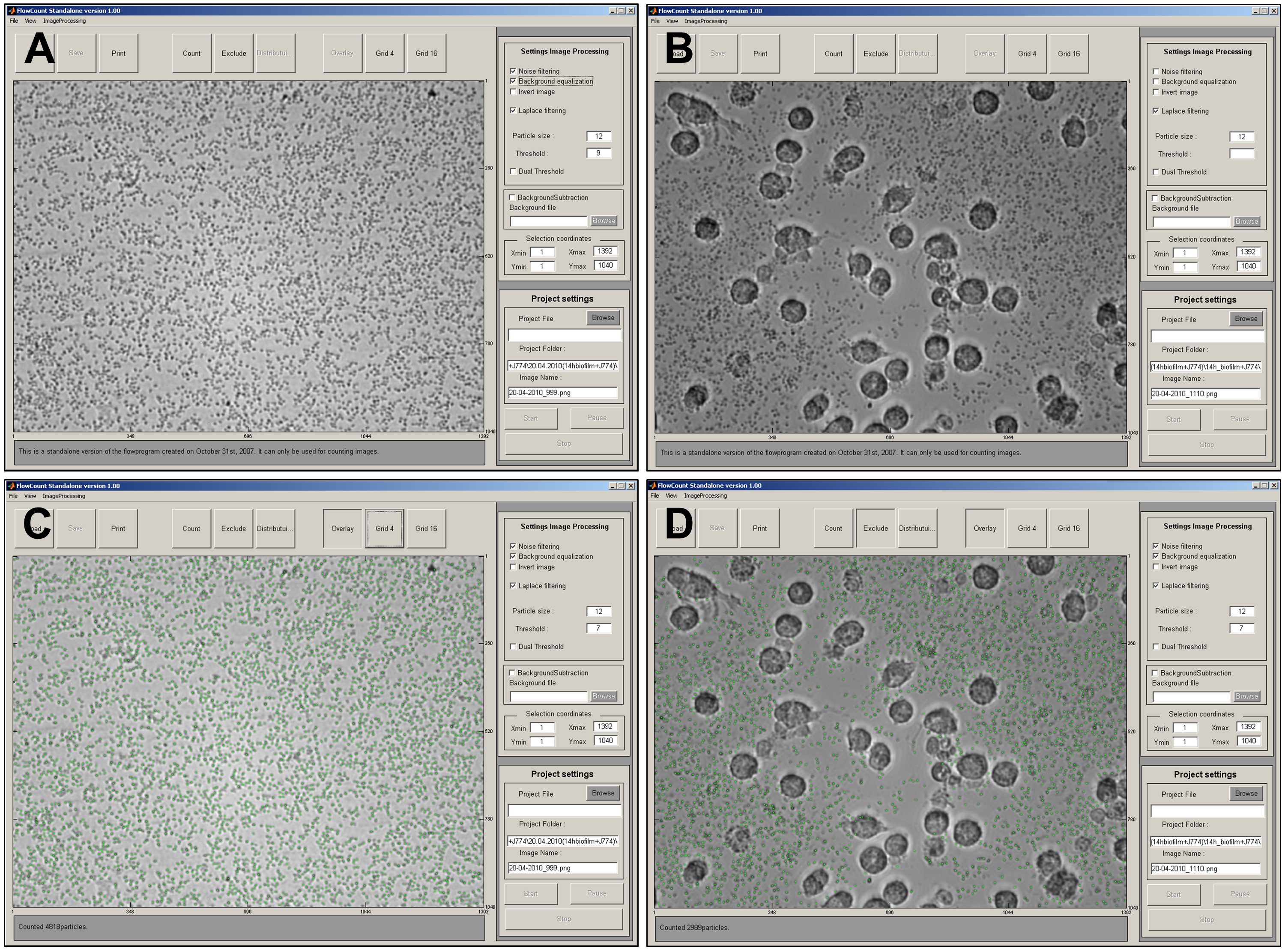

Supplement: Figure S1 — Phase contrast images of adhering bacteria after 14 h of growth and 2 h interaction between bacteria and murine macrophages (J77A.1). A) S. epidermidis 3399 after 14 h of growth and 2 h in contact with macrophage medium in absence of macrophages (control), B) S. epidermidis 3399 after 14 h of growth and 2 h interaction with J774A.1, C) Fig. S1A processed with proprietary software, placing a green dot on top of each bacterium recognized by the software, and D) Fig. S1B processed with proprietary software, placing a green dot on top of each bacterium outside J774A.1. Processed images were used to access the number of adhering bacteria to glass per unit area. One field of view in the above series of images covers a surface area of 6.75×10−4 cm−2, from which by conversion the number of adhering bacteria/cm−2 can be easily calculated for images taken after different growth times (see Figure S2), as summarized in Table S2. The difference between the number of adhering bacteria in the presence and in the absence of phagocytes provides with the total number of phagocytized bacteria. Control experiments in absence of phagocytes allow to account for any bacterial growth occurring during 2 h interaction with phagocytes. Since the total number of bacteria phagocytized depends on the number of phagocytic cells present, we also determined the number of phagocytes adhering per unit area, as described above for the adhering staphylococci (see summary in Table S3). Once knowing the number of phagocytes and phagocytized bacteria per unit area, the numbers of bacteria phagocytized within one phagocytic cell follows (see further Video S1). (TIF) [file pone.0070046.s001.tif]

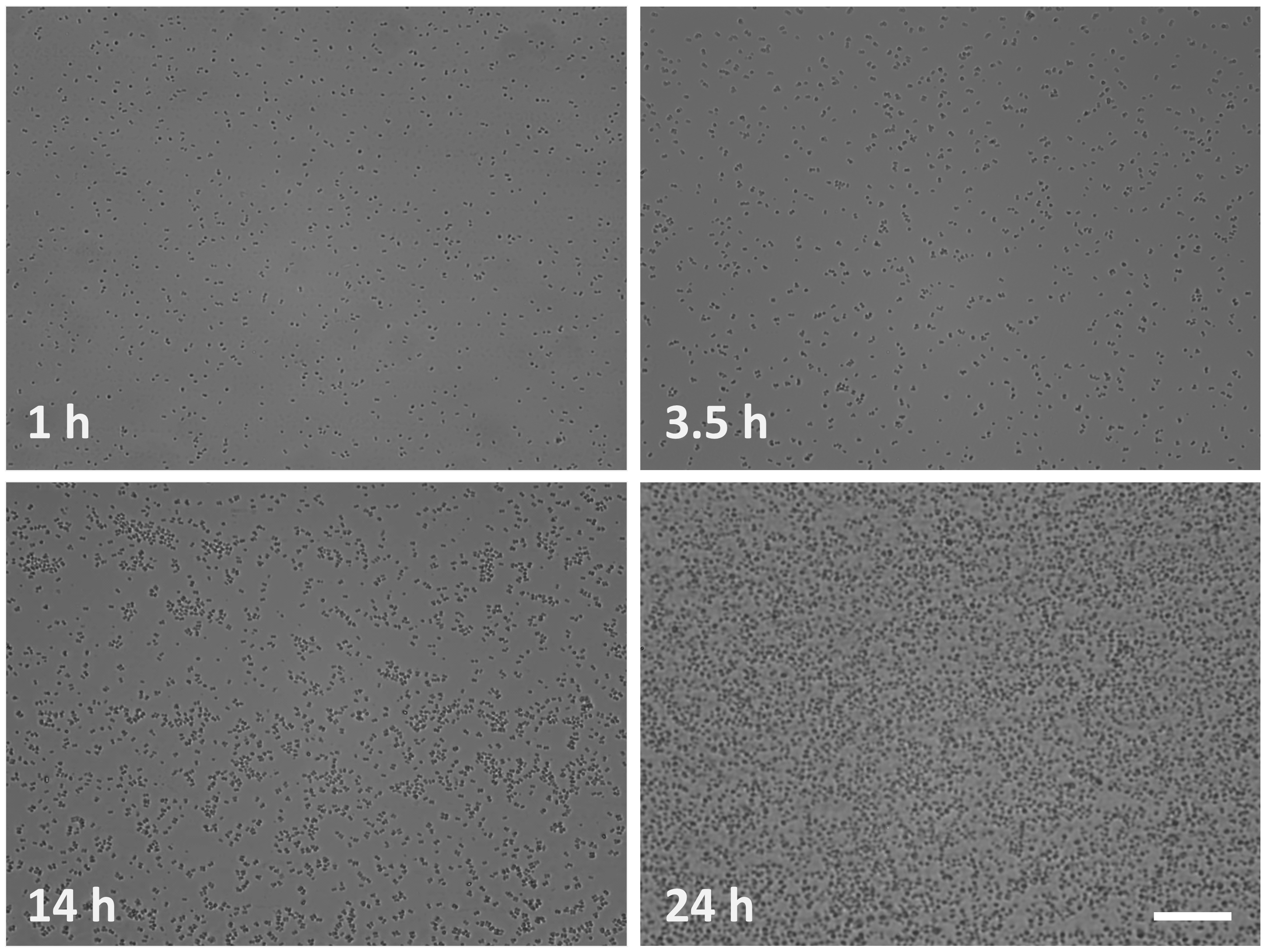

Supplement: Figure S2 — Phase-contrast images of the growth of adhering Staphylococci epidermidis 3399 for different periods of time. Staphylococcal adhesion and growth on a glass surface at 37°C and under constant shear (11 s−1). Scale bar represents 40 µm. (TIF) [file pone.0070046.s002.tif]

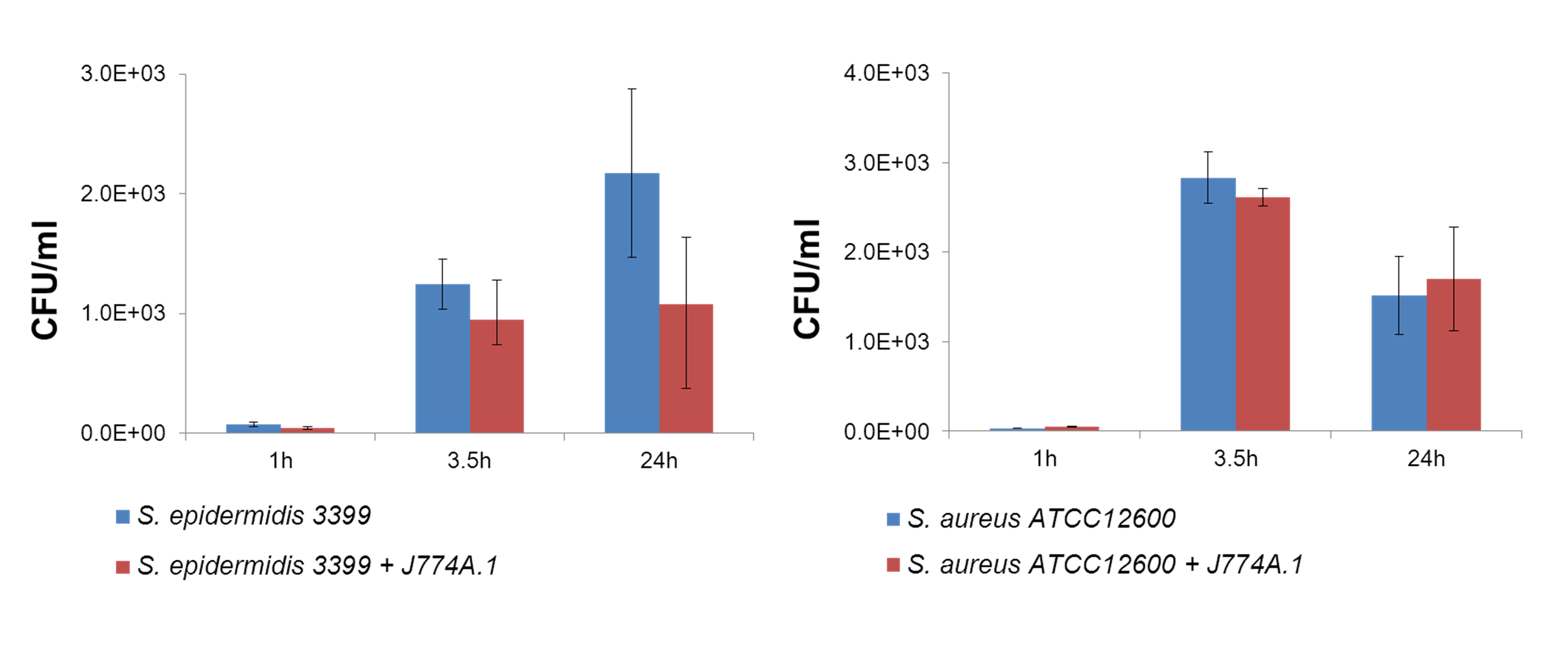

Supplement: Figure S3 — Colony formation units (CFU) per ml of the bacteria grown in solution or detached from the surface after 2 h interaction, in the presence and absence of murine macrophages (J774A.1). No statistical differences were found between the number of bacteria found in solution in the absence and presence of phagocytes. Experiments were done in tissue culture polystyrene well plates (static conditions), where 1 ml of bacterial suspension (1×10^6^ bacteria/ml) was grown for 1 h in tryptone soya broth (TSB) at 37°C. Subsequently non-adhering bacteria were removed by washing three times with DMEM-HG+10%FBS and 1 ml of macrophage suspension (6×10^4^ bacteria/ml) or medium without macrophages (control) was added and incubated for 2 h. After 2 h, the medium in each well, with bacteria-macrophages or bacteria-medium, was diluted 1000× and 100 µl was plated on TSB agar plates. Bacterial colonies were quantified after 24 h incubation to identify the number of bacteria that detach during interaction with and without macrophages. Experiments were done in triplicate. (TIF) [file pone.0070046.s003.tif]
