# Supplementary material for: Phagocytosis of Bacteria Adhering to a Biomaterial Surface in a Surface Thermodynamic Perspective
Source: PLoS One. 2013 Jul 19;8(7):e70046. doi: 10.1371/journal.pone.0070046 (PMC3716708; doi:10.1371/journal.pone.0070046)
Supplement: Table S1 — R2 values of a linear fit through the data describing the number of staphylococci internalized per phagocyte as a function of the number of initially adhering staphylococci. Note, that the linear function was mathematically forced to pass through the origin, i.e. zero staphylococci internalized when the number of initially adhering staphylococci is zero (see Figure 3). (DOC) [file pone.0070046.s004.doc]

**Table S1. R^2^ values of a linear fit through the data describing the number of staphylococci internalized per phagocyte as a function of the number of initially adhering staphylococci.**

Note, that the linear function was mathematically forced to pass through the origin, i.e. zero staphylococci internalized when the number of initially adhering staphylococci is zero (see Figure 3).

|  | **J774A.1** | **THP-1** | **HL-60** |
| --- | --- | --- | --- |
| ***S. epidermidis* 3399** | 0.94 | 0.80 | -0.42 |
| ***S. epidermidis* 7391** | 0.65 | 0.86 | 0.63 |
| ***S. epidermidis* 1457** | 0.97 | 0.73 | 0.71 |
| ***S. aureus* ATCC12600^GFP^** | 0.87 | 0.88 | 0.56 |
| ***S. aureus* 7323** | 0.85 | 0.72 | 0.34 |
| ***S. aureus* LAC** | 0.62 | 0.99 | 0.97 |
