# Supplementary material for: Phagocytosis of Bacteria Adhering to a Biomaterial Surface in a Surface Thermodynamic Perspective
Source: PLoS One. 2013 Jul 19;8(7):e70046. doi: 10.1371/journal.pone.0070046 (PMC3716708; doi:10.1371/journal.pone.0070046)
Supplement: Table S2 — Number of staphylococci (106 bacteria/cm2) adhering to glass after growth for different periods of time, in absence (control) and presence of phagocytes. Data are presented prior to (interaction time 0 h) and after interaction with phagocytes (interaction time 2 h). (DOC) [file pone.0070046.s005.doc]

**Table S2. Number of staphylococci (10^6^ bacteria/cm^2^) adhering to glass after growth for different periods of time, in absence (control) and presence of phagocytes.**

**Data are presented prior to (interaction time 0 h) and after interaction with phagocytes (interaction time 2 h).**

| **InteractionTime (h)** | **Bacterial strains** | **Biofilm growth** | **Control** | **J774A.1** | **THP-1** | **HL-60** |
| --- | --- | --- | --- | --- | --- | --- |
| **0** | ***S. epidermidis* 3399** | **1h** | 1.2 ± 0.6 | 1.7 ±0.4 | 1.1 ± 0.4 | 1.4 ± 0.2 |
|  |  | **3.5h** | 2.5 ± 0.7 | 2.6 ± 0.2 | 2.6 ± 0.2 | 1.5 ± 0.8 |
|  |  | **14h** | 7.0 ± 0.8 | 6.2 ± 1.6 | 6.2 ± 1.6 | 4.1 ± 1.4 |
|  |  | **24h** | 11.4 ± 3.5 | 12.9 ± 6.3 | 12.1 ± 0.9 | 12.3 ± 1.3 |
|  | ***S. epidermidis* 7391** | **3.5h** | 3.0 ± 1.4 | 3.2 ± 0.5 | 4.9 ± 0.7 | 3.8 ± 2.0 |
|  | ***S. epidermidis* 1457** | **3.5h** | 3.3 ± 1.6 | 3.1 ± 0.6 | 4.6 ± 1.6 | 4.7 ± 1.5 |
|  | ***S. aureus* ATCC12600^GFP^** | **1h** | 1.3 ±0.2 | 1.4 ± 0.1 | 1.2 ± 0.1 | 1.1 ± 0.2 |
|  |  | **3.5h** | 10.6 ± 2.7 | 7.0 ± 3.2 | 9.4 ± 4.1 | 8.8 ± 0.9 |
|  | ***S. aureus* 7323** | **3.5h** | 6.3 ± 2.2 | 6.1 ± 1.4 | 7.1 ± 2.2 | 6.8 ± 1.7 |
|  | ***S. aureus* LAC** | **3.5h** | 10.1 ± 5.6 | 11.0 ± 7.0 | 15.6 ± 3.3 | 10.3 ± 3.2 |
| **2** | ***S. epidermidis* 3399** | **1h** | 1.4 ± 0.5 | 0.9 ± 0.4 | 1.0 ± 0.4 | 1.1 ± 0.3 |
|  |  | **3.5h** | 3.5 ± 0.5 | 2.9 ± 0.7 | 4.9 ± 1.8 | 2.2 ± 1.3 |
|  |  | **14h** | 6.7 ± 0.3 | 4.5 ± 0.6 | 5.3 ± 0.8 | 4.7 ± 2.0 |
|  |  | **24h** | 13.4 ± 4.0 | 10.1 ± 5.9 | 9.7 ± 2.2 | 13.0 ± 1.7 |
|  | ***S. epidermidis* 7391** | **3.5h** | 4.1 ± 0.9 | 1.5 ± 0.2 | 3.8 ± 0.4 | 3.8 ±1.2 |
|  | ***S. epidermidis* 1457** | **3.5h** | 3.9 ± 2.2 | 2.3 ± 0.7 | 4.6 ± 1.6 | 4.6 ± 1.4 |
|  | ***S. aureus* ATCC12600^GFP^** | **1h** | 2.2 ± 0.2 | 1.4 ± 0.4 | 1.3 ± 0.3 | 1.5 ± 0.5 |
|  |  | **3.5h** | 14.9 ± 3.2 | 7.9 ± 5.0 | 10.3 ± 6.1 | 10.8 ± 1.3 |
|  | ***S. aureus*** **7323** | **3.5h** | 7.4 ±0.9 | 2.0 ± 1.3 | 5.3 ± 2.1 | 7.7 ± 2.4 |
|  | ***S. aureus* LAC** | **3.5h** | 10.0 ± 9.5 | 9.7 ± 7.1 | 13.3 ± 3.1 | 9.2 ± 2.9 |
