# Supplementary material for: Phagocytosis of Bacteria Adhering to a Biomaterial Surface in a Surface Thermodynamic Perspective
Source: PLoS One. 2013 Jul 19;8(7):e70046. doi: 10.1371/journal.pone.0070046 (PMC3716708; doi:10.1371/journal.pone.0070046)
Supplement: Table S3 — The number of phagocytes (104 phagocytes/cm2) present in the individual experiments. (DOC) [file pone.0070046.s006.doc]

**Table S3. The number of phagocytes (10^4^ phagocytes/cm^2^) present in the individual experiments.**

| **Bacterial strains** | **Biofilm growth** | **J774A.1** | **THP-1** | **HL-60** |
| --- | --- | --- | --- | --- |
| ***S. epidermidis* 3399** | **1 h** | 4.4 ± 1.7 | 4.3 ± 2.1 | 7.0 ± 1.4 |
|  | **3.5 h** | 3.2 ± 2.2 | 4.4 ± 1.6 | 7.6 ± 3.6 |
|  | **14 h** | 4.1 ± 2.2 | 4.2 ± 1.6 | 6.5 ± 2.2 |
|  | **24 h** | 3.1 ± 0.4 | 4.4 ± 2.1 | 6.9 ± 1.1 |
| ***S. epidermidis* 7391** | **3.5 h** | 5.4 ± 2.7 | 6.1 ± 1.2 | 8.1 ± 3.1 |
| ***S. epidermidis* 1457** | **3.5 h** | 5.8 ± 2.8 | 5.1 ± 2.0 | 5.5 ± 1.8 |
| ***S. aureus* ATCC12600^GFP^** | **1 h** | 5.2 ± 1.9 | 5.3 ± 1.8 | 6.6 ± 1.9 |
|  | **3.5 h** | 4.4 ± 1.7 | 5.4 ± 2.1 | 7.6 ± 2.6 |
| ***S. aureus* 7323** | **3.5 h** | 4.3 ± 2.1 | 3.5 ± 2.9 | 8.6 ± 3.7 |
| ***S. aureus* LAC** | **3.5 h** | 7.0 ± 1.0 | 6.7 ± 0.9 | 8.7 ± 0.9 |
